# IL-24 Inhibits Lung Cancer Cell Migration and Invasion by Disrupting The SDF-1/CXCR4 Signaling Axis

**DOI:** 10.1371/journal.pone.0122439

**Published:** 2015-03-16

**Authors:** Janani Panneerselvam, Jiankang Jin, Manish Shanker, Jason Lauderdale, Jonathan Bates, Qi Wang, Yan D. Zhao, Stephen J. Archibald, Timothy J. Hubin, Rajagopal Ramesh

**Affiliations:** 1 Department of Pathology, University of Oklahoma Health Sciences Center, Oklahoma City, Oklahoma, United States of America; 2 Stephenson Cancer Center, University of Oklahoma Health Sciences Center, Oklahoma City, Oklahoma, United States of America; 3 Department of Thoracic and Cardiovascular Surgery, The University of Texas MD Anderson Cancer Center, Houston, Texas, United States of America; 4 Department of Biostatistics and Epidemiology, University of Oklahoma Health Sciences Center, Oklahoma City, Oklahoma, United States of America; 5 Department of Chemistry, The University of Hull, Hull, United Kingdom; 6 Department of Chemistry, Southwestern Oklahoma State University, Weatherford, Oklahoma, United States of America; 7 Graduate Program in Biomedical Sciences, Oklahoma City, Oklahoma, United States of America; University of South Alabama Mitchell Cancer Institute, UNITED STATES

## Abstract

**Background:**

The stromal cell derived factor (SDF)-1/chemokine receptor (CXCR)-4 signaling pathway plays a key role in lung cancer metastasis and is molecular target for therapy. In the present study we investigated whether interleukin (IL)-24 can inhibit the SDF-1/CXCR4 axis and suppress lung cancer cell migration and invasion *in vitro*. Further, the efficacy of IL-24 in combination with CXCR4 antagonists was investigated.

**Methods:**

Human H1299, A549, H460 and HCC827 lung cancer cell lines were used in the present study. The H1299 lung cancer cell line was stably transfected with doxycycline-inducible plasmid expression vector carrying the human IL-24 cDNA and used in the present study to determine the inhibitory effects of IL-24 on SDF-1/CXCR4 axis. H1299 and A549 cell lines were used in transient transfection studies. The inhibitory effects of IL-24 on SDF1/CXCR4 and its downstream targets were analyzed by quantitative RT-PCR, western blot, luciferase reporter assay, flow cytometry and immunocytochemistry. Functional studies included cell migration and invasion assays.

**Principal Findings:**

Endogenous CXCR4 protein expression levels varied among the four human lung cancer cell lines. Doxycycline-induced IL-24 expression in the H1299-IL24 cell line resulted in reduced CXCR4 mRNA and protein expression. IL-24 post-transcriptionally regulated CXCR4 mRNA expression by decreasing the half-life of CXCR4 mRNA (>40%). Functional studies showed IL-24 inhibited tumor cell migration and invasion concomitant with reduction in CXCR4 and its downstream targets (pAKT^S473^, pmTOR^S2448^, pPRAS40^T246^ and HIF-1α). Additionally, IL-24 inhibited tumor cell migration both in the presence and absence of the CXCR4 agonist, SDF-1. Finally, IL-24 when combined with CXCR4 inhibitors (AMD3100, SJA5) or with CXCR4 siRNA demonstrated enhanced inhibitory activity on tumor cell migration.

**Conclusions:**

IL-24 disrupts the SDF-1/CXCR4 signaling pathway and inhibits lung tumor cell migration and invasion. Additionally, IL-24, when combined with CXCR4 inhibitors exhibited enhanced anti-metastatic activity and is an attractive therapeutic strategy for lung metastasis.

## Introduction

Effective treatment of lung cancer remains a challenge with an overall 5-year survival rate of patients diagnosed with lung cancer being less than 16% [1, 2]. One major reason for the dismal survival rate of lung cancer patients is metastasis [3]. Thus, effective control of lung cancer metastasis will reduce the incidence of mortality and increase patient survival.

Signaling between the chemokine receptor CXCR4 and its ligand SDF-1, otherwise known as chemokine ligand (CXCL)-12, contributes to tumor growth, angiogenesis, invasion and metastases in several solid tumors, including non-small cell lung cancer [4]. The interaction between SDF-1 and CXCR4 directs tumor cells to distant organ sites through chemotaxis and homing of metastatic cells [5]. High cell surface expression of CXCR4 has been associated with metastatic activity of tumor cells [6, 7]. Inhibiting the SDF-1/CXCR4 axis with a neutralizing antibody or transfection with an antisense oligonucleotide against CXCR4 significantly reduced invasion, migration and adhesion of lung cancer cell lines *in vitro* [7]. Furthermore, blocking CXCR4 attenuated the aggressiveness of metastasis in non-small cell lung cancer (NSCLC) [4, 8]. Consistent with the pre-clinical study results, clinical studies have shown high CXCR4 expression in NSCLC tumors is associated with metastasis and an increased risk of disease recurrence [9–12]. Molecular studies have shown that SDF-1/CXCR4 axis promotes tumor cell survival, cell migration and metastasis by modulating numerous signaling pathways [13, 14]. Therefore, targeting the SDF-1/CXCR4 axis has received considerable attention for inhibiting tumor metastasis.

Currently AMD3100 (plerixafor, Mozobil) is an FDA approved CXCR4 antagonist that is being tested as a cancer therapeutic [13]. Although AMD3100 has shown efficacy against solid tumors in preclinical studies, the results from clinical studies have not been encouraging [13, 15, 16]. Thus, testing for additional CXCR4 inhibitors that can effectively disrupt the SDF-1/ CXCR4 signaling pathway is warranted.

The human melanoma differentiation associated gene (*mda*)-7/*IL-24* is a unique cytokine/tumor suppressor gene that belongs to the IL-10 cytokine family [17]. Endogenous IL-24 protein expression is detectable in the peripheral blood mononuclear cells (PBMCs), T- and B-cells and in melanocytes [18–20]. However, IL-24 protein expression is lost in a majority of cancer cells of human origin [17]. Studies by Ellerhorst et al., [21] and Ishikawa et al., [22] showed that loss of IL-24 expression correlated with disease progression in melanoma and lung cancer respectively indicating a tumor suppressive role for IL-24. Pre-clinical studies showed that exogenous expression of human IL-24 in a broad spectrum of human cancer cell lines resulted in potent anti-tumor and anti-metastatic activity both *in vitro* and *in vivo* [23–25]. Further, the utility of IL-24 as an anti-cancer drug was demonstrated in a Phase I clinical trial using adenovirus- *mda-7* (INGN-241)-based cancer gene therapy approach [26]. While mda-7/IL-24 is being developed as a cancer therapeutic, the molecular mechanisms by which it exerts it anti-tumor and anti-metastatic activities are not completely understood.

In the present study, we investigated the ability of IL-24 to inhibit the SDF-1/CXCR4 signaling pathway. The rationale to test the IL-24 inhibitory activity on SDF-1/CXCR4 axis and its consequence on cell migration and invasion stems from our recent observation showing that IL-24 inhibited the AKT/mTOR pathway [27]. Since AKT/mTOR is downstream of CXCR4 and is involved in the SDF-1/CXCR4 signaling pathway, we hypothesized that IL-24 regulates cell migration and invasion by disrupting the SDF-1/CXCR4 axis in NSCLC. Additionally, we hypothesized that IL-24 when combined with CXCR4 antagonists (AMD3100, SJA5) would exhibit enhanced anti-metastatic activity.

We demonstrate that (i) IL-24 inhibits lung tumor cell migration and invasion by disrupting the SDF-1/CXCR4 signaling pathway and (ii) IL-24, when combined with CXCR4 antagonists or siRNA, exhibits enhanced anti-metastatic activity. Thus, combining IL-24 with CXCR4 inhibitors is an attractive therapeutic strategy for controlling lung cancer metastasis.

## Methods

### Cell culture

Human non-small cell lung cancer cell (NSCLC) lines were maintained as previously described [25, 28].

### Stable transfection of inducible IL-24 plasmid vector in H1299 cells

Human IL-24 cDNA previously cloned in pLJ143 plasmid backbone was released from a pLJ143 plasmid by restriction enzyme digestion and was recloned into the pTET-ON plasmid vector (Clonetech, Mountain View, CA, USA). Cloning of the IL-24 cDNA at the appropriate restriction enzyme site of the pTET-ON plasmid was confirmed by restriction enzyme digestion and DNA sequencing. The resulting plasmid labeled as pTET-IL-24 was then propagated in E. coli (DH5α strain) and purified using Qiagen Maxi Kit (Qiagen, Valencia, CA, USA) per manufacturer recommendations.

IL-24 protein expression upon addition of doxycycline (1 μg/ml) was determined by conducting a transient transfection assay in H1299 cells using Fugene (Roche, Indianapolis, IN, USA). After confirming that doxycycline induced IL-24 protein expression, we used the pTET-IL-24 plasmid for generating a Tet-inducible stable cancer cell line. Briefly, H1299 cells seeded in six-well plates were transfected with the pTET-IL24 plasmid DNA (1 μg) mixed with Fugene in serum free RPMI medium. At twenty-four hours after transfection, G418 (800 μg/ml; Sigma Chemicals, St. Louis, MO, USA) was added to the wells and the cells were selected for fourteen days. The surviving cells were selected, expanded and screened for doxycycline-induced IL-24 expression by Western blotting. Cell population that showed IL-24 protein expression were subsequently subjected to single cell clonal expansion and screened for IL-24 protein expression. The clone that demonstrated the highest IL-24 protein expression upon addition of doxycycline was labeled as H1299-IL24 and was used in our studies.

### Cell migration assay

A cell migration assay using polycarbonate filters with a pore size of 8 μm (BD Biosciences, Bedford, MA, USA) was performed as previously described [28]. Briefly, H1299-IL24 (5 x 10^4^) cells were seeded in the upper chamber of the insert and placed in a six-well plate filled with serum free RPMI-1640 medium (lower chamber). After 24 h, the culture medium in the six-well plate was replaced with fresh medium containing 20% tetracycline free FBS (Atlanta Biologicals, Inc., Flowery Branch, GA, USA) and the upper chamber was filled with 2% tetracycline free FBS containing medium with or without doxycycline (1μg/ml; Sigma Chemicals). Following incubation for 6 h, 24 h and 48 h, the inserts were removed and processed as previously described. The results were expressed as an average number of migrated cells per microscopic field.

To determine the inhibitory effect of IL-24 against exogenous SDF-1 induced tumor cell migration, a cell migration assay was performed as described above except that the lower chamber contained SDF-1 (100 ng/ml) instead of 20% FBS.

For determining the combined inhibitory effect of IL-24 and AMD3100, cells suspended in 2% tetracycline free FBS containing medium were seeded in the upper chamber of the inserts and were treated with doxycycline alone (1 μg/ml), AMD3100 (100 ng/ml) alone or a combination of both. The culture medium in the lower chamber contained SDF-1 (100 ng/ml). Cells that did not receive any treatment served as a control in these experiments. At 24 h after treatment, the number of migrated cells was counted as described above.

For studies testing the combinatorial inhibitory activity of IL-24 with SJA5 on cell migration in the presence of SDF-1 (100 ng/ml), cells (5 x 10^4^) were treated either with doxycycline (1 μg/ml) or SJA5 (100 ng/ml) or AMD3100 (100 ng/ml) as a single agent or in combination of doxycycline +SJA5 or doxycycline +AMD3100. Cells that did not receive any treatment served as a control. The number of migrated cells was determined at 24 h of treatment as described above.

### Cell invasion assay

A Matrigel cell invasion assay was performed as previously described [29]. Matrigel pre-coated filters (8 μm; BD Biosciences) were rehydrated with 1 ml of tissue culture medium. H1299-IL24 (5 x 10^4^) cells were seeded in the upper chamber, whereas the lower chamber of the insert was filled with serum free tissue culture medium. After 24 h, the lower chamber was replaced with 20% tetracycline free FBS containing medium and the upper chamber was filled with 2% tetracycline free FBS with or without doxycycline (1 μg/ml). Following incubation for an additional 6 h, 24 h and 48 h, the chambers were processed and the tumor cell invasiveness was determined as previously described.

### Flow cytometric analysis

H1299-IL24 cells (1 x 10^6^) were either not treated (control) or treated with SDF-1 (100 ng/ml). At 1 h, 4 h, and 24 h after SDF-1 treatment the cells were harvested, dissociated into single cells, and were washed two times with PBS-BSA buffer. Cells were then fixed for 20 min in 1 ml PBS containing 1% paraformaldehyde at room temperature. After rinsing in PBS-BSA buffer, the cells were incubated with 10% human AB serum (Cat.No.110-HG-100; R & D Systems, Minneapolis, MN, USA) for 20 min at 4°C. Upon completion of the incubation the cells were washed two times with PBS-BSA and then stained with mouse anti-human CXCR4 PE-conjugated monoclonal antibody (Cat.No.FAB170P; 10 μl/10^6^ cells; R & D Systems) for 30 min at 4°C in the dark. The cells were subsequently processed and analyzed for CXCR4 expression using a flow cytometer (FACS Calibur; Becton Dickinson, San Jose, CA, USA).

### Luciferase reporter assay

H1299-IL24 cells (1x10^5^) seeded in six-well tissue culture plates were transiently transfected with 2 μg of pORF9-hCXCR4 Quanti-Luc plasmid (InvivoGen, San Diego, CA, USA) encapsulated in cationic DOTAP:Cholesterol liposome [30]. After 6 h of transfection, tissue culture medium was removed and replenished with fresh medium supplemented with or without doxycycline (1 μg/ml). At 24 h after doxycycline treatment, 10 μl of culture supernatant was taken from each sample and transferred to a 96-well white (opaque) plate and 50 μl of Quanti-Luc assay (InvivoGen) reagent was added and luciferase activity was measured by Perkin Elmer EnVision Multilabel Reader (Waltham, MA, USA), according to the manufacturer’s instruction. The results from duplicate wells for each sample was calculated and represented as the average of duplicate samples. Experiments were performed independently for a minimum of three times for calculating statistical significance.

### Real-time-PCR analysis and measurement of RNA stability

H1299-IL24 cells seeded in six-well plates were treated with doxycycline (1 μg/ml). At 6 h and 24 h after doxycycline treatment the cells were harvested and used for total RNA isolation. Cells that were not treated with doxycycline served as control. Total RNA from the control and doxycycline-treated H1299-IL24 cells was isolated using Trizol (Life technologies, Grand Island, NY, USA) and was subjected to reverse transcription using iScript cDNA synthesis kit (Bio-Rad, Hercules, CA, USA) and the complementary DNA (cDNA) was subsequently used to perform real-time (RT)-PCR (Bio-Rad CFX96 TouchReal-Time PCR Detection System) with SYBR chemistry using iQTM SYBR Green super mix (Bio-Rad) and using human CXCR4-specific oligonucleotide primers (Forward-5’CCACCATCTACTCCATCATCTTC 3’-Sense, Reverse-5’ACTTGTCCGTCATGCTTCTC3’-AntiSense) (Integrated DNA Technologies, Coralville, IA, USA). Thermal cycling was programmed as follows: 95°C for 30 s followed by 40 cycles of 95°C for 20s, 62°C for 20 s and 72°C for 20 s. The crossing threshold (Ct) value assessed by RT-PCR was noted for the transcripts and normalized with human 18S mRNA (Forward- 5’-CAGCCACCCGAGATTGAGCA-3’ and Reverse- 5’-TAGTAGGGACGGGCGGTGTG-3’) (Integrated DNA Technologies). The changes in mRNA were expressed as fold change relative to control ± the standard deviation (SD).

To determine the effect of SDF-1 treatment on CXCR4 expression, H1299-IL24 cells were harvested for total RNA isolation at 30 min, 1 h, 6 h and 24 h after SDF-1 (100 ng/ml) treatment. Cells that were not treated with SDF-1 served as the control. RT-PCR using human CXCR4 specific oligonucleotide primers was performed as described above.

To determine the stability of CXCR4 mRNA, cells (1 x10^5^) were treated with or without doxycycline (1 μg/ml) for 24 h. The following day, the cells were treated with actinomycin D (3 μM; Amersco LLC, Solon, OH, USA) and were harvested at 30 min, 1 h, 2 h, 3 h, 4 h, 6 h and 24 h after actinomycin D treatment. Total RNA prepared from the harvested cells was used to determine the CXCR4 mRNA levels by RT-PCR as described above. CXCR4 mRNA half-lives were calculated from typical decay curves by linear regression between 0 h and 24 h [31]. Values ± SD are based on at least two independent experiments

### Transient transfection assay

H1299 and A549 cells (1 x 10^5^) were seeded in six-well tissue culture plates and transiently transfected with 1 μg of plasmid expression vector carrying the *IL-24* cDNA and encapsulated in cationic DOTAP:Cholesterol liposome as previously described [30]. After 6 h of transfection, tissue culture medium was aspirated and replenished with fresh medium. Cells that were not transfected with the *IL-24* plasmid vector served as control. The cells were harvested at 24 h after transfection, cell lysate prepared and analyzed for IL-24, CXCR4, and AKT by western blotting.

### Western blotting assays

Cells receiving various treatments and collected at various time points were subjected to Western blot analysis as previously described [32]. Primary antibodies against IL-24 (1:2000; Introgen Therapeutics, Houston, TX, USA), GRK6 (SC-100380; 1:1000; Santa Cruz Biotechnology, Inc., California, CA, USA), phospho-CXCR4^S339^ (ab74012) and CXCR4 (ab2074) (1:1000; Abcam, Cambridge, MA, USA), phospho-CXCR4^S324/325^ (CP 4251; 1:1000; ECM Biosciences LLC, Versailles, KY, USA), phospho-AKT^S473^ (Cat. No. 4060), total AKT (Cat. No. 9272), phospho-PRAS40^T246^ (Cat. No. 2997), total PRAS40 (Cat. No. 2691), phospho-mTOR^S2448^ (Cat. No. 2971) and total mTOR (Cat. No. 2983) (1:1000; Cell Signaling Technology Inc; Beverly, MA, USA), CXCR7 (PA5-28739; 1: 1000; Thermo Scientific; Rockford, IL, USA); HIF-1α (ABE 279; 1:1000; Millipore), Beta actin (1:2000; Sigma Chemicals) were purchased and used as recommended by the manufacturers. Proteins were detected using appropriate secondary antibodies (Santa Cruz Biotechnology, Inc., and Jackson ImmunoResearch Laboratories, Inc., West Grove, PA, USA) and an enhanced chemiluminescence kit (Thermo Scientific). Protein levels were detected using chemiluminescence imaging system (Syngene, Frederick, MD) and quantified using Image Quant (Syngene) software.

### Immunocytochemistry

Cells (1.0 x 10^4^) were seeded on Lab-Tek 2-well chamber slides (Nalge-Nunc International, Rochester, NY, USA) and were either not treated (control) or treated with doxycycline (1 μg/ml). At 24 h after treatment, the cells were processed and stained as previously described [25, 28, 30]. Primary antibodies used were mouse anti-human IL-24 antibody (1:1000) and rabbit anti-human CXCR4 antibody (1:2000). The slides were cover-slipped, observed, and photographs captured using Nikon TiU microscope (Nikon Instruments Inc. Melville, NY, USA).

### Calcium mobilization assay

Cells grown on poly-D-lysine coated plates were treated with doxycycline (1 μg/ml) for 24 h. Cells that were not treated with doxycycline served as the control. The following day, the cells were labeled with Fluo-4 Direct calcium reagent (Molecular probes, Eugene, Oregon, USA) by adding the reagent to the tissue culture medium as recommended by the manufacturer’s protocol, and incubating for 1 h at 37°C. SDF-1 (100 ng/ml) was then added in all of the wells, and the cytosolic free calcium concentration was determined at different time points by measuring the fluorescence (excitation at 495 nm and emission at 516 nm) using Perkin Elmer EnVision Multilabel Reader (Waltham, MA, USA). The results were plotted against time and expressed as relative fluorescence units (RFU).

### CXCR4 RNA interference studies

Cells (1 x 10^5^ cells/well) were seeded in 6 well plates and transfected with 100 nM of CXCR4 siRNA (Santa Cruz Biotechnology) using DOTAP:Cholesterol liposome [30]. Six-hours after transfection in serum free medium, the tissue culture medium was replaced with 2% tetracycline free serum containing RPMI-1460 and SDF-1 (100 ng/ml). The cells were then either not treated or treated with doxycycline (1 μg/ml). Cells that were not transfected with siRNA served as a control. After 24 h of incubation, the cells were harvested and total cell lysate prepared and analyzed for CXCR4, AKT, and PRAS40 by Western blotting.

For the cell migration assay, cells (5 x 10^4^) were seeded in the upper chamber of the migration inserts and were transfected with CXCR4 siRNA (100 nM) and were either not treated or treated with doxycycline (1 μg/ml). The lower chamber was filled with SDF-1 (100 ng/ml) containing tissue culture medium. The number of migrated cells at 24 h after doxycycline treatment was counted as described above.

### Statistical analysis

Unless otherwise stated, all data were shown as mean ± standard deviation of the mean (SD). Univariate statistical significance was determined by one-way analysis of variance (ANOVA) with Tukey’s adjustment for pairwise comparisons. Difference between groups at each time point was obtained using linear mixed effects model with Tukey’s adjustment. A p-value of less than 0.05 was considered statistically significant. SAS 9.2 was used for the statistical analyses.

## Results

### NSCLC cells express CXCR4 and AKT

**Fig 1:**
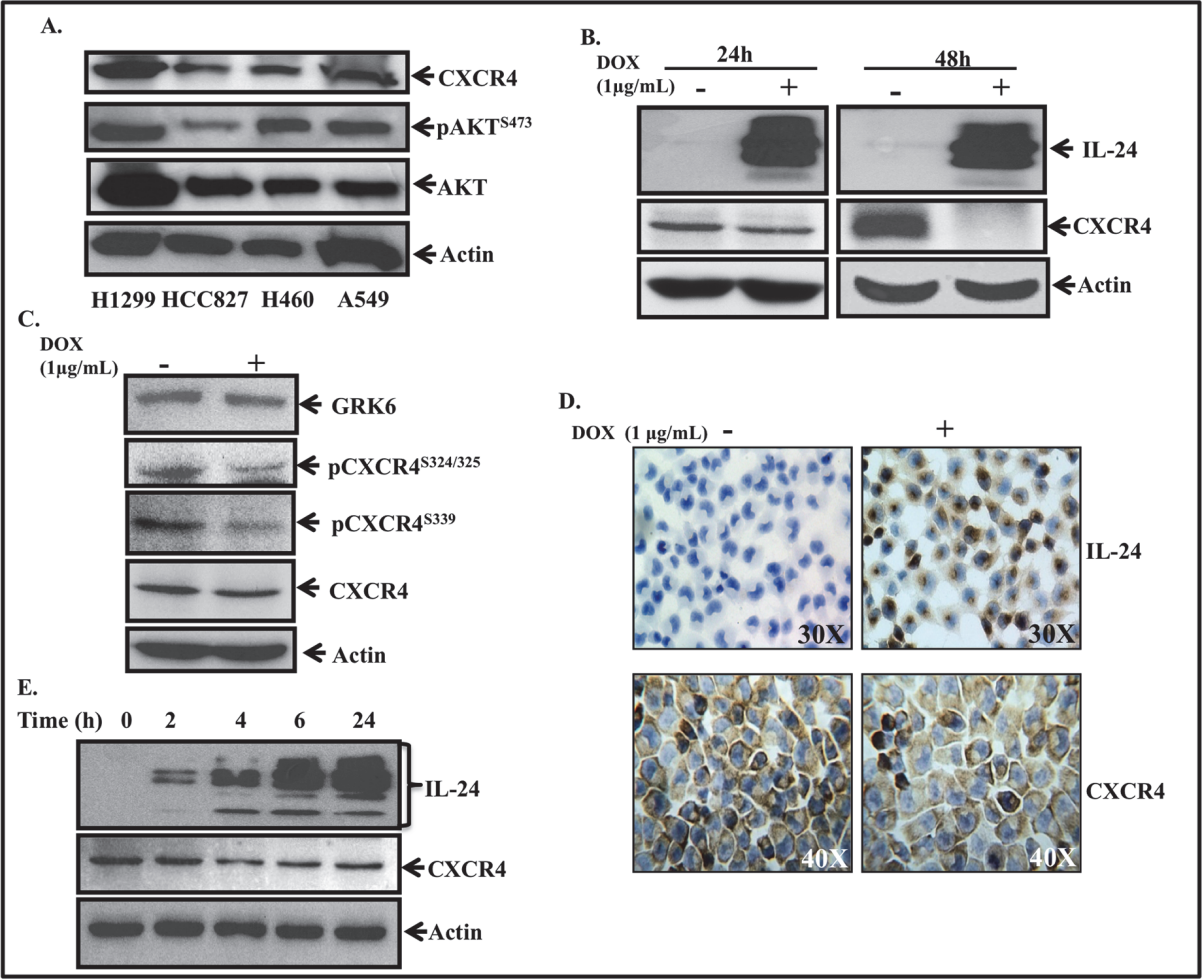
CXCR4 expression in human lung cancer cells and its inhibition by IL-24. ***A*,** Endogenous CXCR4 and AKT protein expression in human lung cancer cell lines. ***B*,** IL-24 reduced CXCR4 expression at 24 h and 48 h in doxycycline-treated H1299-IL24 cells but not in doxycycline untreated control cells. ***C*,** IL-24 reduced GRK6, phosphorylated (p) CXCR4 and total CXCR4 expression in doxycycline-treated H1299-IL24 cells compared to expression of these proteins in doxycycline untreated H1299-IL24 cells. ***D***, Immunocytochemistry showing doxycycline-induced IL-24 expression in H1299-IL24 cell line reduced CXCR4 expression. Cells that were not treated with doxycycline served as control. *Magnification* IL-24- X 30; CXCR4- X 40. ***E*,** Time-course study showed CXCR4 expression was reduced as early as 4 h after IL-24 expression and the inhibitory activity was sustained up to 24 h in doxycycline-treated H1299-IL24 cells. Beta actin was used as protein loading control in Western blotting assays.

Expression levels of endogenousCXCR4 and AKT (phosphorylated AKT^S473^ and total AKT) proteins varied among the four human lung cancer (H1299, HCC827, H460, and A549) cell lines tested with H1299 cell line showing the highest expression level of the two proteins (Fig. 1A). Based on our findings, we chose to use the H1299 cell line for all of our studies described below.

### IL-24 downregulates CXCR4 but not CXCR7 expression

To determine the inhibitory activity of IL-24 on CXCR4, we first generated an IL-24 inducible cell line of H1299 that was transfected with a doxycycline inducible plasmid vector (pTET-*IL-24*) and selected to express IL-24 on addition of doxycycline. The cell line thus created was labeled “H1299-IL24” and used in the present study. Note: H1299 cells do not express endogenous IL-24 protein and hence any IL-24 protein detected on addition of doxycycline is attributed to the induction of the IL-24.

Treatment of H1299-IL24 cells with doxycycline (1 μg/ml) resulted in IL-24 expression at 24 h and 48 h (Fig. 1B). Associated with IL-24 expression was a marked reduction in CXCR4 expression at both time points tested (Fig. 1B). Since activation of the CXCR4 pathway involves CXCR4 phosphorylation (p) at Serine 324/325 and Serine 339 by the G protein coupled receptors (GPCRs) kinase (GRK)-6 [33–35], we examined the inhibitory effect of IL-24 on GRK6, pCXCR4^S324/325^ and pCXCR4^S339^. IL-24 reduced the expression of GRK6, pCXCR4^S324/325^, pCXCR4^S339^ and total CXCR4 expression (Fig. 1C). Immunocytochemical staining showed doxycycline-treated H1299-IL24 cells expressed IL-24 and had reduced CXCR4 (Fig. 1D) compared to cells that were not treated with doxycycline (control). This observation concurred with our Western blotting result. We next conducted a time-course study to determine how early IL-24 expression could reduce CXCR4 expression. IL-24 protein expression was detectable as early as 2 h after doxycycline treatment, and its expression increased over time (Fig. 1E). In parallel, inhibition of CXCR4 was observed to occur starting at 4 h and the inhibitory activity was sustained to 24 h of testing (Fig. 1E). These results showed that IL-24 effectively inhibited CXCR4 expression in a time-dependent manner.

To determine if the observed IL-24-mediated inhibition on CXCR4 was unique to H1299 cells, we conducted experiments in an additional lung cancer cell line, A549. H1299 and A549 cells were transiently transfected with an IL-24 expressing plasmid DNA vector and the cell lysates collected at 24 h after transfection were analyzed by Western blotting. Cells that were not transfected with the plasmid vector served as control. IL-24 expression was detectable in both the cell lines that were transfected with the IL-24 plasmid DNA while no IL-24 protein expression was detected in the control cells (S1 Fig.). Associated with IL-24 expression was a marked reduction in CXCR4 protein expression in both the cell lines. Additionally, reduction in phosphorylated AKT^S473^, a downstream target of CXCR4 was also observed in cells expressing IL-24, but not in control cells. Our study results demonstrate that IL-24-mediated inhibitory activity on CXCR4 is not restricted to one cell line and that the inhibitory activity on CXCR4 can be achieved via inducible or transient IL-24 expression.

Since SDF-1 can bind to both CXCR4 and CXCR7, we determined CXCR7 expression in lung cancer cell lines and whether IL-24 inhibited CXCR7 in H1299-IL24 cell line. Endogenous CXCR7 protein expression levels varied among the lung cancer cell lines tested (S2 Fig.). However, induction of IL-24 expression in H1299-IL24 cell line did not inhibit CXCR7 (S2 Fig.) compared to control cells. Our study results thus demonstrate that IL-24 selectively inhibits CXCR4 and not CXCR7 in H1299 cells.

### IL-24-mediated CXCR4 inhibition results in reduced tumor cell migration and invasion

**Fig 2:**
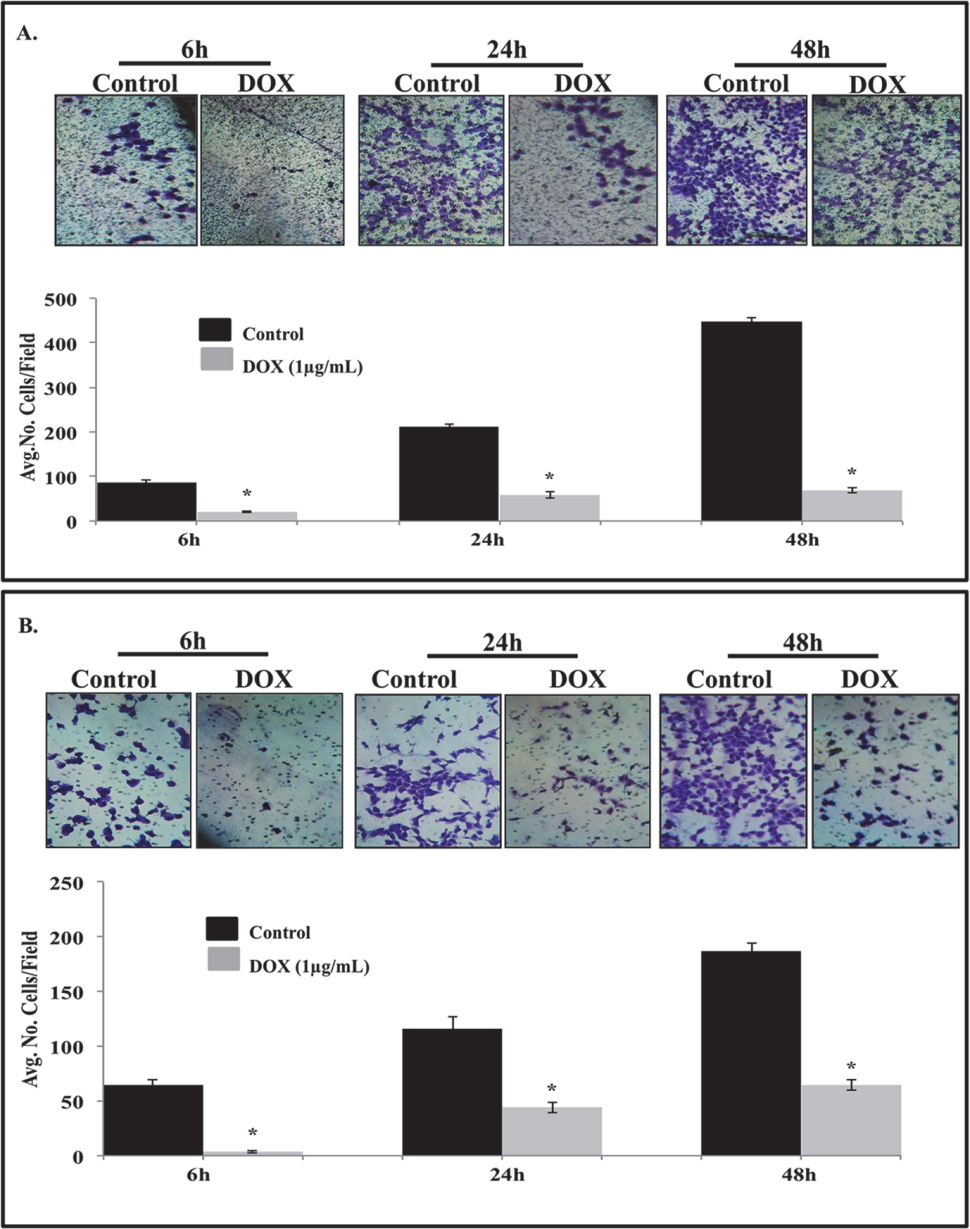
IL-24 suppresses lung cancer cell migration and invasion. H1299-IL24 cells were either not treated or treated with doxycycline and observed for cell migration and invasion. IL-24 inhibited tumor cell migration (A) and invasion (B) starting from 6h and sustained its inhibitory activity till 48 h at which time point the experiment was terminated (*P*<0.05). Bars denote standard deviation (SD).

Since CXCR4 has been shown to play a role in tumor metastasis by promoting cell migration and invasion we investigated whether the attenuation of CXCR4 expression by IL-24 had a consequential biological effect. IL-24 induction in H1299-IL24 cells significantly reduced cell migration as early as 6 h and was sustained until 48 h when compared to the control (Fig. 2A; *P*<0.05). The possibility that the inhibitory activity was due to cell killing was eliminated as no cytotoxicity was observed at 6 h, an observation that concurred with our previous studies using adenovirus (Ad)-IL-24 [25].

The possibility that doxycycline treatment alone could produce a direct inhibitory effect on cell migration [36] was also excluded by treating naïve H1299 cells with doxycycline (1 μg/ml). Doxycycline treated naïve H1299 cells demonstrated no inhibitory effect on cell migration when compared to control cells (S3 Fig.).

A tumor cell invasion assay also showed that IL-24 significantly reduced the number of cells invading through Matrigel compared to control cells at all-time points tested (Fig. 2B; *P*<0.05). Our results indicate that disruption of CXCR4 signaling by IL-24 results in inhibition of both tumor cell migration and invasion.

### Inhibition of CXCR4 by IL-24 affects AKT-mTOR signaling

**Fig 3:**
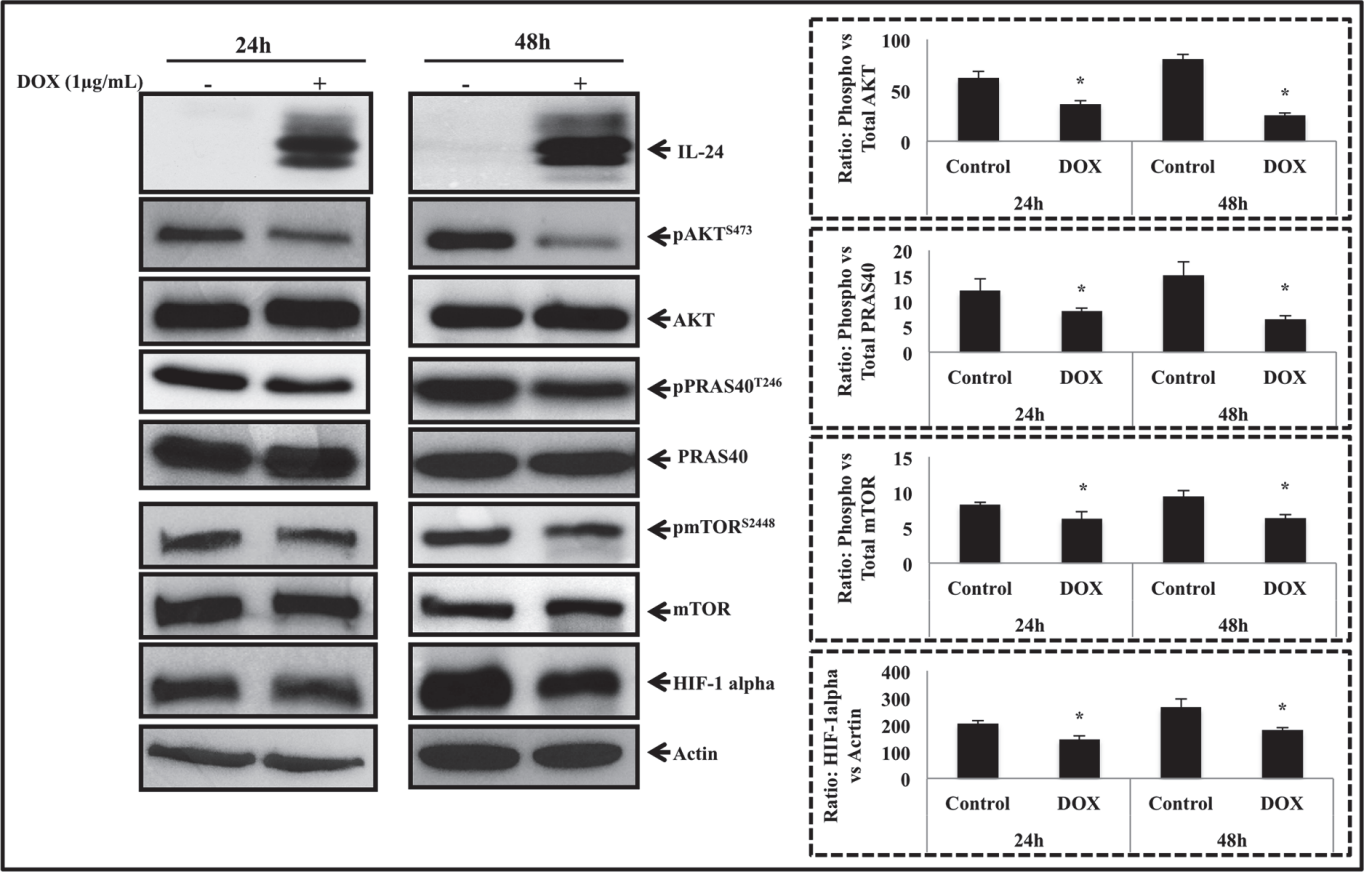
IL-24 inhibited the signaling proteins downstream of CXCR4. Western blotting showed induction of IL-24 protein expression in H1299-IL24 cells resulted in marked reduction in the expression of phosphorylated (p) AKT^S473^, pmTOR^S2448^ and pPRAS40^T246^ and HIF-1α at 24 h and 48 h after doxycycline treatment. Beta actin was used as protein loading control. Differences in the expression of the proteins was determined by semi-quantitative analysis and represented in graphical format (*P*<0.05). Bars denote standard deviation (SD).

Expression and activation of CXCR4 has been shown to positively regulate several developmental and oncogenic signaling pathways in many cancer types [13, 37, 38]. Recent evidence shows that the SDF-1/CXCR4 axis and the PI3K/AKT axis functionally interact and play a significant role in tumor growth, metastasis and therapy resistance [39]. Additionally, the AKT/mTOR pathway is downstream of CXCR4 and has been shown to be regulated by CXCR4 activation [40]. Since IL-24 inhibited CXCR4 and consequently inhibited cell migration and invasion, we next studied the inhibitory effects of IL-24 on the AKT/mTOR pathway. Expression of IL-24 in H1299-IL24 cells resulted in a marked reduction in pAKT^S473^ protein expression compared to control cells at both 24 h and 48 h (Fig. 3; *P*<0.05). Concurrent with reduction in pAKT^S473^ expression was the reduced expression of pPRAS40^T246^, which is a direct substrate of AKT. Furthermore, expression of pmTOR^S2448^ and HIF-1α was also markedly reduced with IL-24 induction (Fig. 3; *P*<0.05). These data demonstrate that IL-24-mediated CXCR4 inhibition effectively reduces expression of signaling molecules that are downstream of CXCR4 and are involved in tumor cell migration and invasion.

### CXCR4 is regulated at the post-transcriptional level by IL-24

**Fig 4:**
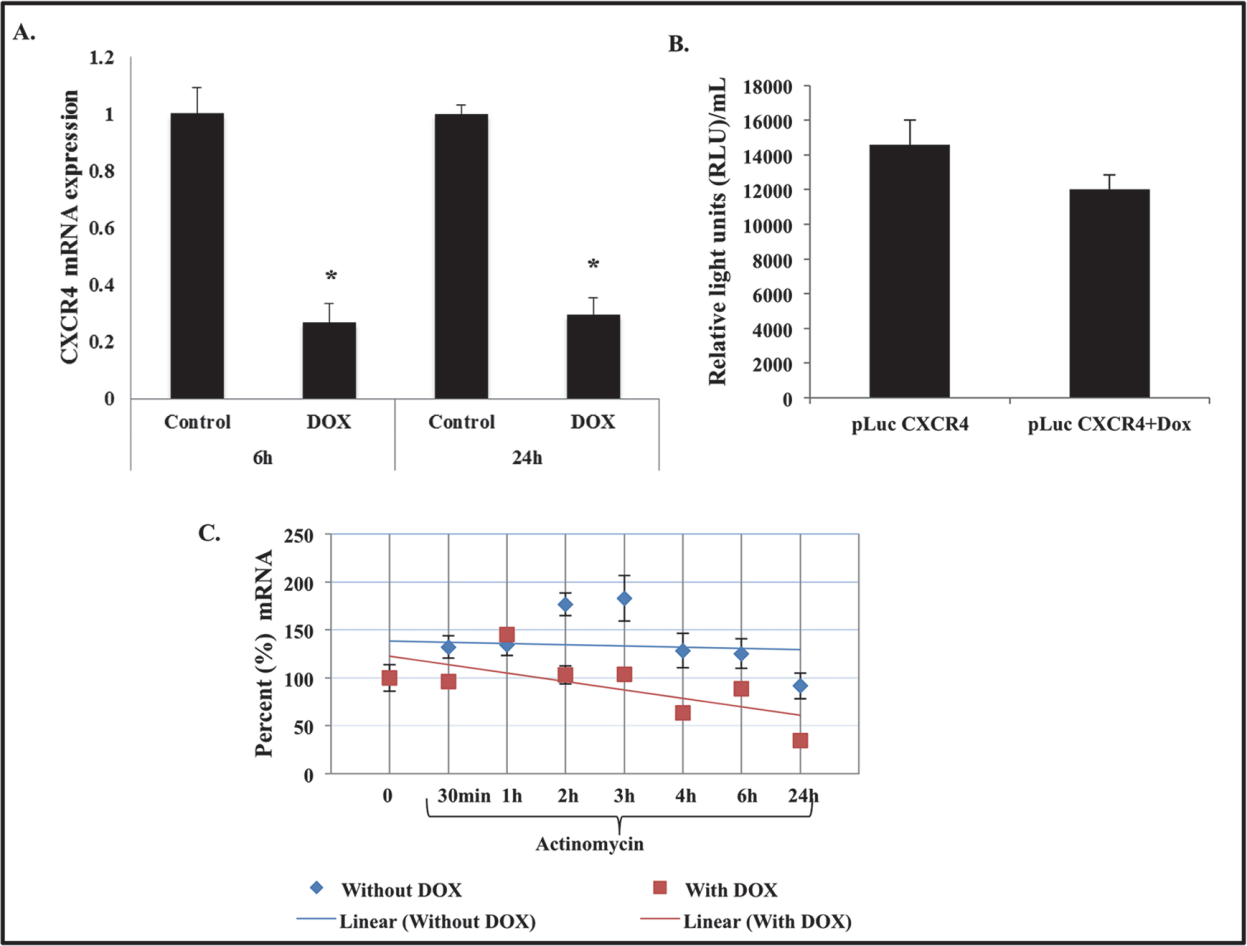
IL-24 regulated CXCR4 at post-transcriptional level. ***A*,** RT-PCR analysis showed IL-24 reduced CXCR4 mRNA levels at 6 h and 24 h (*P*<0.05). ***B*,** CXCR4 promoter activity was determined using a luciferase reporter vector. Induction of IL-24 showed no significant reduction in luciferase activity indicating IL-24 did not affect the CXCR4 promoter. ***C*,** mRNA stability studies showed IL-24 reduced the half-life of CXCR4 mRNA at approximately 4 h. Bars denote standard deviation (SD).

We next determined whether IL-24 regulated CXCR4 at the transcriptional level. IL-24 induction in H1299-IL24 cells significantly reduced CXCR4 mRNA expression at 6 h and 24 h when compared to the control (Fig. 4A; *P*<0.05). To elucidate the mechanism of how IL-24 regulated CXCR4 mRNA expression, we analyzed the promoter activity in H1299-IL24 cells that was transiently transfected with a luciferase reporter vector driven by the human CXCR4 promoter region of 279 base pairs. IL-24 failed to inhibit the CXCR4 promoter activity as evidenced by the lack of significant reduction in luciferase reporter activity (Fig. 4B). This result indicated that the IL-24 did not regulate CXCR4 mRNA expression at the promoter level.

We next speculated that IL-24 might regulate CXCR4 mRNA by altering the mRNA stability and consequently protein expression. To test this possibility, cells were pretreated with doxycycline for 24 h and subsequently treated with or without (control) the transcription inhibitor, actinomycin D. At different time points after actinomycin D treatment, the cells were collected and analyzed for CXCR4 mRNA expression. We observed that the CXCR4 mRNA expression was significantly reduced (>40% reduction over control) at 4 h when IL-24 expression was induced compared to CXCR4 mRNA expression in control cells (Fig. 4C). Our data showed that IL-24 regulates CXCR4 at the post-transcriptional level by reducing its stability.

### IL-24 inhibits SDF-1-induced CXCR4 molecular signaling and tumor cell migration

Prior to testing the inhibitory activity of IL-24 on SDF-1 induced CXCR4 signaling, we determined whether H1299-IL24 cells responded to SDF-1. SDF-1 is the known ligand for CXCR4 receptor activation [41]. For this purpose, cells were either not treated or treated with recombinant SDF-1 (100 ng/ml) and analyzed for CXCR4 mRNA expression by qRT-PCR and for CXCR4 expression at the cell surface by flow cytometric analysis.

**Fig 5:**
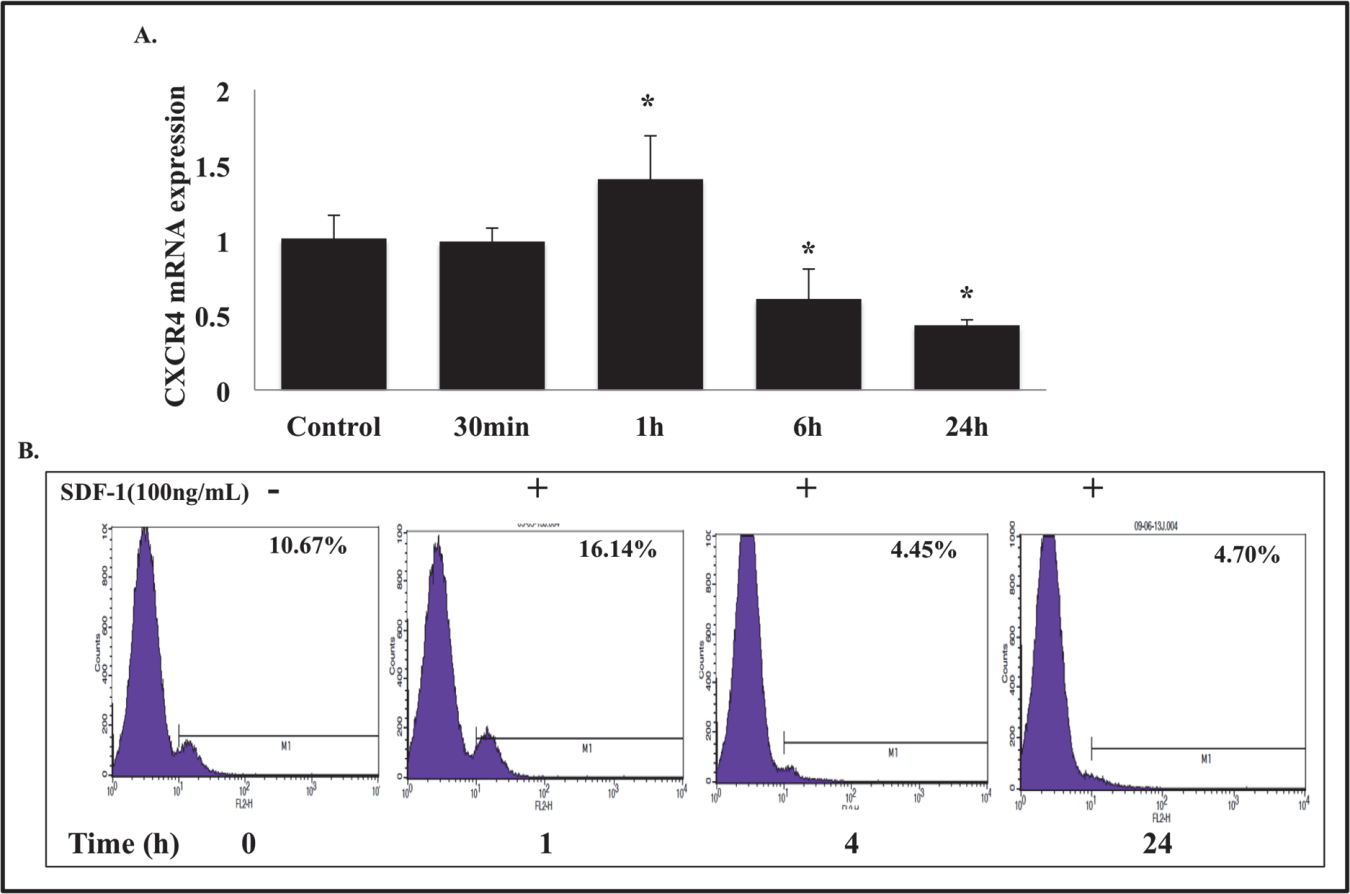
SDF-1 mediated CXCR4 activation in H1299 cells. ***A*,** RT-PCR studies and ***B***, Flow cytometry analysis showed SDF-1 activated CXCR4 mRNA and cell surface expression in H1299-IL24 cell line indicating CXCR4 is functionally active and intact (*P*<0.05). Bars denote standard deviation (SD).

SDF-1 maximally induced CXCR4 mRNA expression at 1 h after treatment followed by a decrease at later time points (Fig. 5A). Analysis for cell surface expression showed the highest CXCR4expression (16.14% positive; Fig. 5B) at 1 h after SDF-1 treatment compared to control cells (10.67% positive). After 1 h, the CXCR4 expression at the cell surface decreased (4.45% and 4.70% at 4 h and 24 h respectively) and was markedly lower than the expression in the control cells. The lower CXCR4 expression levels at later time points suggested that SDF-1 induced CXCR4 is likely endocytosed from the cell surface for receptor degradation or recycling back to the cell surface [42]. Our data nevertheless showed that the SDF-1 activity on CXCR4 was rapid and occurred within the first 1 h of treatment in H1299-IL24 cells.

In a separate experiment, we determined the intracellular calcium level as a measure of SDF-1/CXCR4 interaction [41] in the presence and absence of IL-24 expression.SDF1 treatment resulted in mobilization of the intracellular calcium pool and release of [Ca^2+^] that increased over time (S4 Fig.) in control (without IL-24 induction) cells. In contrast, IL-24 expression resulted in marked suppression of SDF-1-mediated mobilization of the intracellular calcium pool and [Ca^2+^] release starting at 30 min after SDF-1 treatment (S4 Fig.). The highest inhibitory activity, however, was observed from 3 h to 6 h. Although the IL-24-mediated inhibitory activity was not statistically significant compared to the control, it definitely showed a trend for continued inhibition on [Ca^2+^] release over time indicating abrogation of SDF-1/CXCR4 signaling.

**Fig 6:**
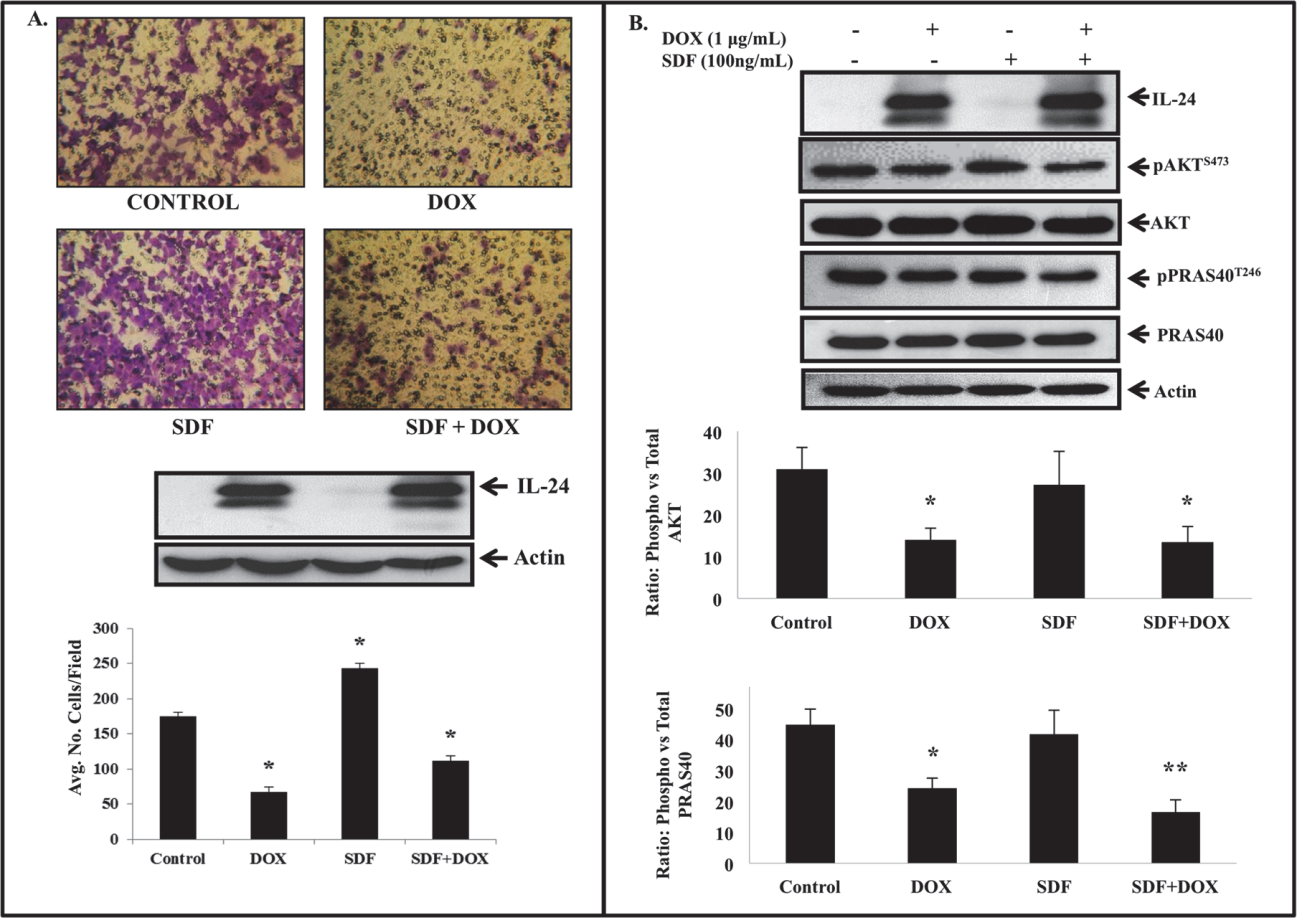
IL-24 suppresses SDF-1/CXCR4 signaling and tumor cell migration. ***A*,** IL-24 significantly inhibited tumor cell migration in the presence and absence of SDF-1. The inhibitory activity in the presence of SDF-1 however was less than that observed in the absence of SDF-1 (*P*<0.05). Error bars denote standard deviation. ***B*,** Induction of IL-24 protein expression in H1299-IL24 cells resulted in marked reduction in pAKT^S473^ and pPRAS40^T246^ protein expression at 24 h after doxycycline treatment. Beta actin was used as protein loading control. Differences in the expression of the proteins was determined by semi-quantitative analysis and represented in graphical format (*P*<0.05). Bars denote standard deviation (SD).

We next investigated whether IL-24 could inhibit SDF-1-induced tumor cell migration and suppress the CXCR4 signaling pathway. SDF-1 alone significantly increased H1299-IL24 cell migration when compared to control cells that did not receive SDF-1 (*P*<0.05; Fig. 6A). In contrast, IL-24 significantly inhibited tumor cell migration both in the absence and presence of SDF-1(Fig. 6A; *P*<0.05). However, the inhibitory activity of IL-24 on SDF-1-induced cell migration was less than the inhibitory activity observed for IL-24 in the absence of SDF-1. Molecular studies showed that IL-24 suppressed SDF-1-induced cell migration by disrupting the AKT/mTOR signaling pathway [43, 44], as evidenced by the reduction in pAKT^S473^ and pPRAS40^T246^ protein expression both in the presence and absence of SDF-1 when compared to control cells and cells that were treated with SDF-1 alone (Fig. 6B; *P*<0.05). The IL-24-mediated inhibitory effect on pPRAS40^T246^ expression but not on pAKT^S473^ expression was statistically significant both in the presence and absence of SDF-1. Our results demonstrate IL-24 effectively inhibited SDF-1/CXCR4 signaling pathway by disrupting the AKT/mTOR signaling.

### IL-24 shows greater inhibitory activity on CXCR4 signaling and tumor cell migration when combined with pharmacologic or genetic inhibitor of CXCR4

AMD3100 is a selective and potent CXCR4 antagonist that efficiently inhibits CXCR4 signaling by interfering with the SDF-1/CXCR4 interaction [45]. Since IL-24 inhibited SDF-1/CXCR4 signaling, we tested the combinatorial inhibitory effect of IL-24 and AMD3100 on SDF-1-induced CXCR4 signaling and H1299-IL24 cell migration.

**Fig 7:**
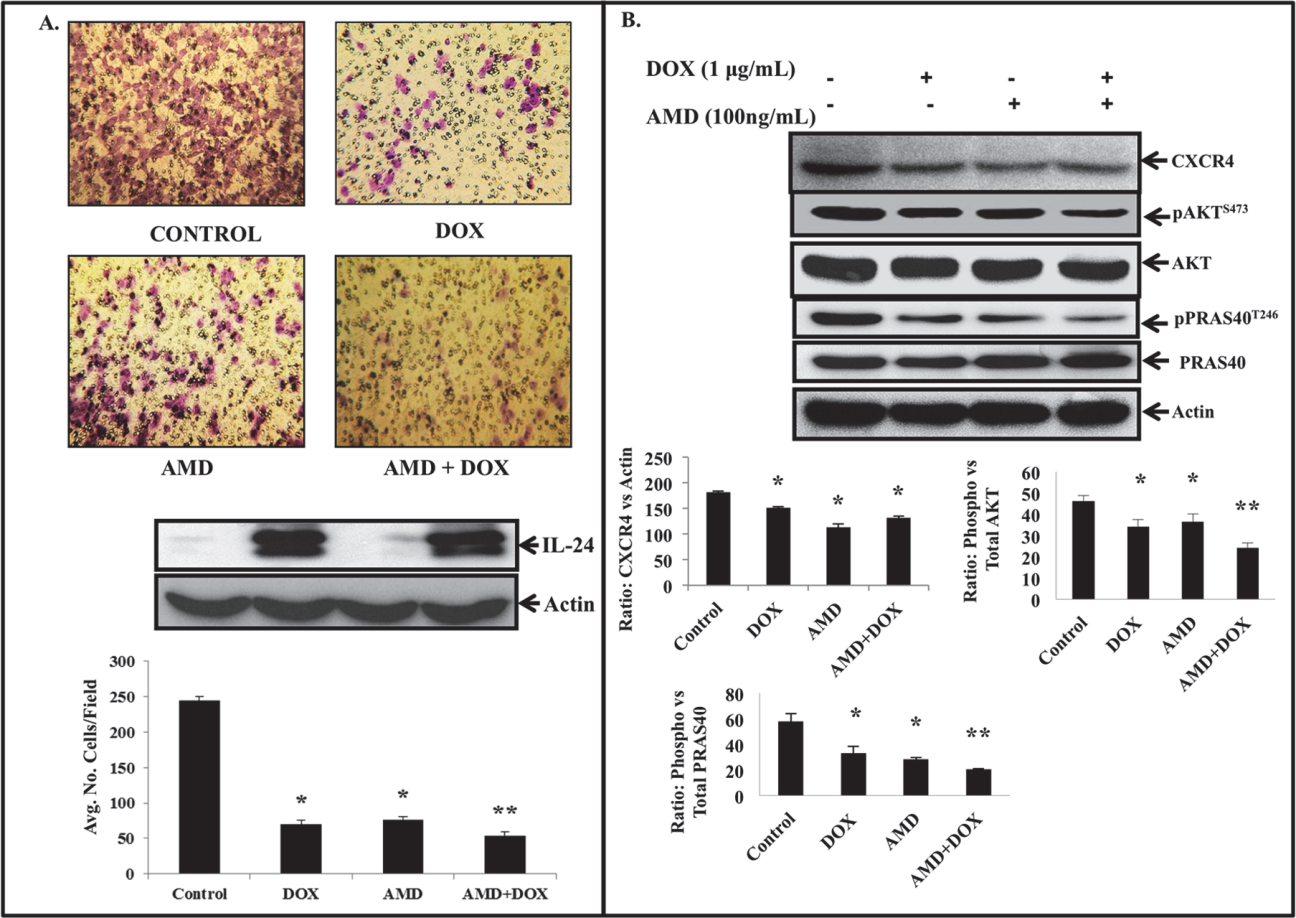
IL-24 combined with AMD3100 exhibited greater inhibitory activity on SDF-1 induced cell migration and SDF-1/CXCR4 signaling. ***A*,** Combination of IL-24 and AMD3100 significantly suppressed SDF-1 induced cell migration compared to number of cells that migrated in the control group (*P*<0.05). Inhibitory effects by IL-24 treatment alone and AMD3100 treatment alone were also significant when compared to the control group. ***B*,** CXCR4 expression was markedly reduced in IL-24 expressing cells, cells treated with AMD3100, and in combination treatment of IL-24 and AMD3100 when compared to control cells. Greater reduction in the expression of pAKT^S473^ and pPRAS40^T246^ proteins were observed in combination treatment groups when compared to all other groups. Beta actin was used as protein loading control. Differences in the expression of the proteins was determined by semi-quantitative analysis and represented in graphical format (*P*< 0.05). Bars denote standard deviation (SD).

SDF-1-induced chemotactic activity of tumor cells was markedly reduced in the presence of AMD3100 or IL-24 alone when compared to control cells (Fig. 7A; *P*<0.05). More importantly, the inhibitory activity on tumor cell migration was greatly enhanced when AMD3100 was combined with IL-24 (Fig. 7A). Correlating with the tumor cell migration study results, CXCR4, pAKT^S473^ and pPRAS40^T246^ protein expression were all reduced in the cells that were treated with AMD3100 alone, IL-24 alone, and AMD3100 plus IL-24 when compared to control cells (Fig. 7B; *P*<0.05). However, a higher inhibitory activity on pAKT^S473^ and pPRAS40^T246^ protein expression was observed in cells that were treated with AMD3100 plus IL-24 when compared to all other treatments. These results demonstrate that combining AMD3100 with IL-24 is more effective in inhibiting the SDF-1/CXCR4 signaling axis and cell migration than either treatment alone.

We also tested another CXCR4 antagonist, SJA5 whose inhibitory activity is equivalent to or greater than AMD3100. One advantage of SJA5 over AMD3100 is that the binding of SJA5 to CXCR4 has been shown to be prolonged compared to AMD3100 [46]. Thus, we tested the inhibitory activity of SJA5 on CXCR4 and compared it with the inhibitory activity of AMD3100 on CXCR4. SJA5 showed greater inhibitory activity on CXCR4 than AMD3100 when compared to control at various time points tested (S5 Fig.; *P*<0.05). Furthermore, combination treatment of SJA5 with IL-24 showed the highest inhibitory activity on tumor cell migration when compared to all other treatment groups including AMD3100 plus IL-24 treatment (S6 Fig.; *P*< 0.05). These results indicate combination of SJA5 and IL-24 will be a better cancer therapeutic.

**Fig 8:**
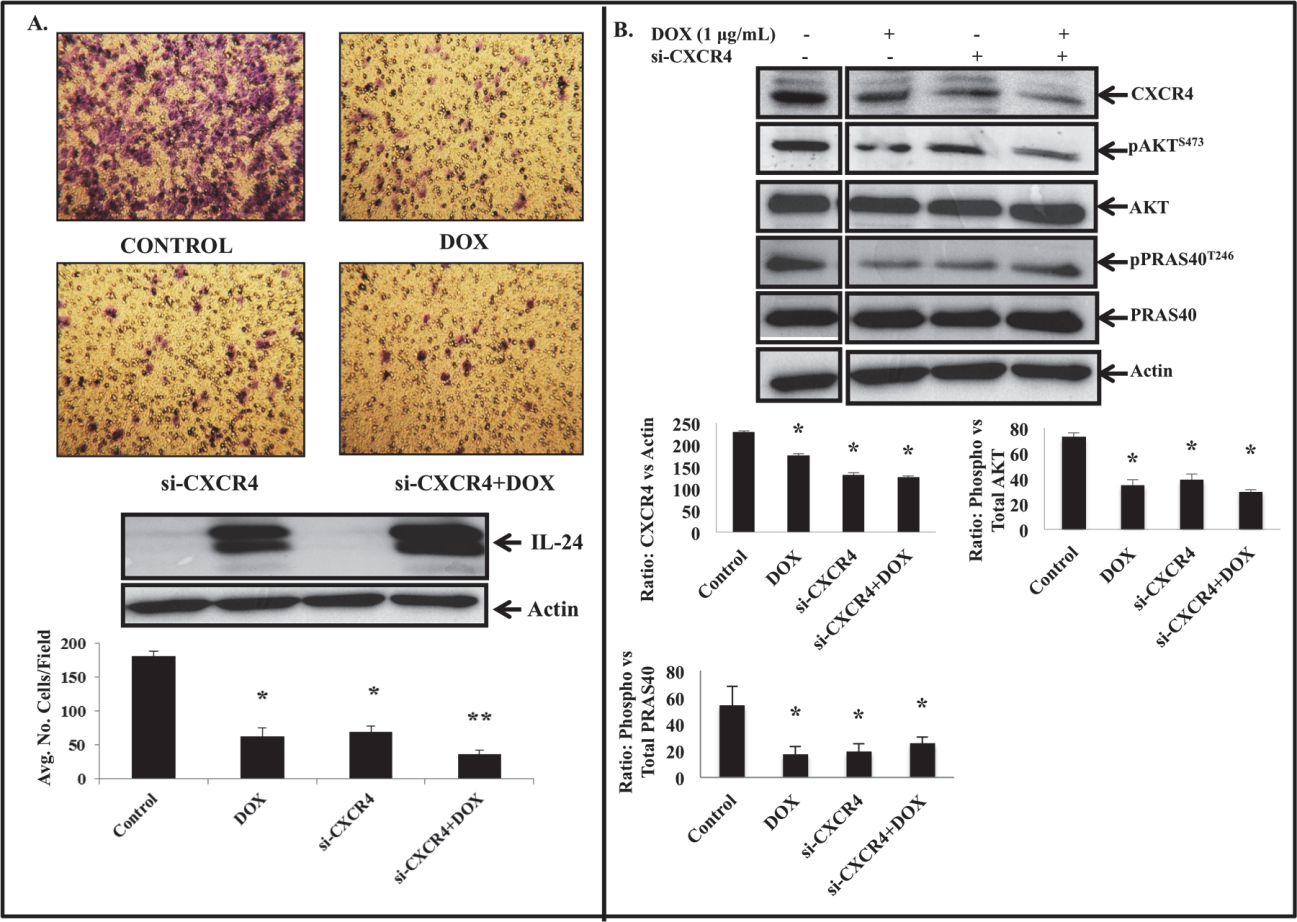
siRNA mediated CXCR4 inhibition in combination with IL-24 produced greater inhibitory activity on tumor cell migration. ***A*,** siRNA-mediated CXCR4 knockdown when combined with IL-24 resulted in a significant suppression of SDF-1 induced tumor cell migration compared to cell migration in the control group (*P*<0.05). Inhibitory effects on tumor cell migration mediated by IL-24 treatment alone and CXCR4 siRNA treatment alone were also significant when compared to control group. ***B*,** Western blotting showed combination of CXCR4 siRNA and IL-24 produced greater reduction in the expression of CXCR4 and pAKT^S473^ proteins when compared to all other groups. pPRAS40^T246^ protein expression was reduced in all treatment groups when compared to control. However, its expression was slightly higher in the combination treatment group when compared to individual treatments. Beta actin was used as protein loading control. Differences in the expression of the proteins was determined by semi-quantitative analysis and represented in graphical format (*P*<0.05). Bars denote standard deviation (SD).

We next investigated whether genetic knockdown of CXCR4 using siRNA would produce an inhibitory effect on SDF-1/CXCR4 signaling and cell migration similar to that observed with AMD3100. siRNA mediated knockdown of CXCR4 reduced tumor cell migration against SDF-1 gradient (Fig. 8A; *P*<0.05). The inhibitory activity observed was comparable to that observed with IL-24 treatment alone. However, when CXCR4 siRNA was combined with IL-24, a significant reduction in cell migration that was higher than all other treatment groups was observed (Fig. 8A; *P*<0.001). Molecular analysis showed CXCR4, pAKT^S473^ and pPRAS40^T246^ protein was significantly (Fig. 8B; *P*<0.05) reduced when compared to control. However, no significant difference in the protein expression levels was observed between IL-24, siRNA, and IL-24 plus siRNA treatments. These results indicate that a siRNA-based therapeutic in combination with IL-24 can be another approach for targeting the SDF-1/CXCR4 signaling axis.

In summary, our study results demonstrate IL-24 disrupts the SDF-1/CXCR4 signaling pathway resulting in reduced cell migration and invasion and combination therapy of IL-24 with pharmacologic or genetic CXCR4 inhibitor produced a greater inhibitory activity on tumor cell migration. Our data provides evidence that IL-24 in combination with CXCR4 inhibitors will be more effective in controlling cancer metastasis.

## Discussion

Studies have demonstrated that the process of tumor metastasis mimics specific mechanisms that are found in normal physiological processes, such as the leukocyte trafficking and homing orchestrated by the chemokine system [47, 48]. Among the several chemokine receptors known to be expressed in several different tumor cells, the CXCR4 chemokine receptor has been shown to play an important role in cancer metastasis [49–53]. Interaction of the SDF-1 ligand with CXCR4 triggers molecular events that favor cell migration, invasion, and metastasis. Thus, disruption of the SDF-1/CXCR4 axis is likely to reduce metastasis, making this is promising target for cancer treatment [53]. While CXCR4 targeted drugs have been developed and are currently in clinical testing, AMD3100 is the only CXCR4 antagonist approved by the FDA for cancer treatment [54, 55]. Clinical study results show that AMD3100 is not effective in controlling tumor metastasis, which warrants the development and testing of additional drugs.

In this study, we demonstrated that IL-24-mediated its anti-metastatic activity by disrupting the SDF-1/CXCR4 axis in lung cancer cells. We showed that the IL-24-mediated inhibitory activity on CXCR4 was comparable when IL-24 was stably induced or expressed transiently in the cancer cell lines and was independent of the lung cancer cell line used. Furthermore, we identified that IL-24 also exerted its inhibitory effect on the internalization and activation of the CXCR4 receptor by attenuating GRK6 and GRK6-mediated phosphorylation of CXCR4.

Studies have shown that CXCR7 can serve as an alternative for CXCR4 and augment SDF-1 mediated G-protein signaling [56, 57]. CXCR7 has been shown to a play a role in the regulation of angiogenesis, stem cell trafficking and cancer metastases [58, 59]. CXCR7 expression in human lung cancer cells has also been reported [58]. These reports invoked us to ask whether IL-24 could also regulate SDF-1/CXCR7 mediated signaling in lung cancer cells. IL-24 did not inhibit CXCR7 expression in H1299-IL24 cells demonstrating that IL-24 specifically regulated SDF-1/CXCR4 signaling and not SDF-1/CXCR7 signaling. On the basis of these observations, we focused our studies in investigating the molecular mechanism by which IL-24 suppressed SDF-1/CXCR4 signaling.

Molecular studies showed that IL-24 did not repress the CXCR4 mRNA by regulating at the promoter level; instead it reduced the stability of the CXCR4 mRNA. PCR studies showed that IL-24 reduced CXCR4 mRNA levels by greater than 40% (*P<0*.*05*) by 4 h in the presence of actinomycin D, an inhibitor of nascent mRNA synthesis. This observation indicated that IL-24 reduced the half-life of CXCR4 mRNA and thus modulated protein expression. The functional consequence of reduced CXCR4 mRNA and protein expression was the significant reduction (*P<0*.*05*) in the migratory and invasive properties of the lung tumor cells. In fact, the inhibitory activity on tumor cell migration and invasion was detectable as early as 6 h indicating that the IL-24-mediated inhibitory activity was not due to cytotoxicity as our previous study using Ad-IL-24 showed measurable cytotoxicity starting only at 24 [25]. This observation correlated with the observed reduction in CXCR4 protein as early as 4 h after IL-24 expression. Thus, there appears to be a very good correlation between IL-24 expression and CXCR4 suppression.

Apart from measuring the IL-24 inhibitory effect on cell migration and invasion, we also investigated whether the AKT/mTOR signaling pathway that is downstream of SDF-1/CXCR4 axis and essential for lung cancer progression and metastasis was also affected [14, 43, 44, 60–62]. Our studies showed that IL-24 effectively suppressed AKT/mTOR signaling (*P<0*.*05*) that culminated in inhibition of tumor cell migration and invasion. Further, the IL-24-mediated inhibitory activity (*P<0*.*05*) was observed even in the presence of SDF-1 demonstrating its potent anti-metastatic activity. To our knowledge, this is the first report demonstrating that IL-24 attenuated the SDF-1/CXCR4 signaling axis in lung cancer cells.

SDF-1 binding with CXCR4 has also been shown to activate signal transduction and activator of transcription (STAT) 3 and that STAT-3 is required for cell migration [63]. Thus, it is plausible that the observed inhibitory activity on cell migration and invasion in part occurred via IL-24 directly inhibiting STAT-3. However, studies from our laboratory and others have previously shown that the antitumor activity of IL-24 occurred independent of STAT-3 [64, 65]. Thus, IL-24 mediated inhibitory effect on cell migration and invasion observed in the present study was due to STAT-3 inhibition could be excluded. Whether IL-24 inhibited additional signaling proteins that play a role in cell migration and invasion has not been investigated and is beyond the scope of the present study.

Studies using AMD3100 have shown that the SDF-1/CXCR4 axis can be effectively inhibited resulting in anti-metastatic activity [66–68]. However, clinical studies have shown AMD3100 not to be very effective, which warrants combination therapy [69]. Therefore, we tested the combined inhibitory activity of IL-24 and AMD3100. Combinatorial studies showed AMD3100 plus IL-24 produced a marked inhibitory effect on SDF-1/CXCR4 signaling and cell migration (*P<0*.*05*). The combination therapy was more effective when compared with either AMD3100 or IL-24 treatment alone (*P<0*.*05*). These results indicate that combination therapy for treatment of metastatic lung cancer is likely to be more effective than individual treatments. However, it will be important to conduct *in vivo* studies to determine the combinatorial therapy efficacy in controlling metastasis.

As an alternate to AMD3100 therapy we have tested SJA5 that has been shown to have improved efficacy in inhibiting CXCR4 [46]. Our results demonstrated that the inhibitory activity exhibited by SJA5 on CXCR4 was greater than the inhibitory activity exhibited by AMD3100. Additionally, combination therapy of IL-24 with SJA5 was more effective in inhibiting tumor cell migration compared to combination therapy of IL-24 and AMD3100 (*P<0*.*05*).

As a final proof that IL-24 mediated its anti-metastatic effects through CXCR4 inhibition, we conducted siRNA-based studies. siRNA mediated CXCR4 knock-down resulted in inhibition of tumor cell migration and was associated with reduced expression of pAKT^S473^ and pPRAS40^T246^ proteins (*P<0*.*05*). Further, the anti-metastatic activity observed when siRNA was combined with IL-24 was comparable to that observed when IL-24 was combined with AMD3100. These results clearly demonstrate that the SDF-1/CXCR4 axis is specifically inhibited by IL-24 and that combination therapy is more effective than individual treatments. Our study also demonstrated that IL-24-based therapy can be combined with different CXCR4 inhibitors to effectively disrupt the SDF-1/CXCR4 signaling axis. Thus, incorporating IL-24 with SDF-1/CXCR4-targeted therapies will be effective in controlling cancer cell metastasis.

## Conclusions

In conclusion we have demonstrated that IL-24 exerts its anti-metastatic activity by disrupting the SDF-1/CXCR4 axis and that IL-24-based therapy in conjunction with CXCR4 inhibitors will be more effective in attenuating lung cancer metastasis. While testing of IL-24 in combination with CXCR4 inhibitors *in vivo* is important, they are outside the scope of the present study. Demonstration of *in vivo* efficacy will advance the development of IL-24/CXCR4 based combinatorial therapeutic interventions for lung cancer.

## Supporting Information

S1 Fig IL-24 inhibits CXCR4 and its downstream target in H1299 and A549 cells. Transient transfection of IL-24 plasmid DNA reduced CXCR4 and pAKT^S473^ protein expression in both H1299 and A549 cells compared to their respective non-transfected cells. Beta actin was used as protein loading control.
(TIF)

S2 Fig IL-24 does not inhibit CXCR7 expression. ***A*,** Western blotting showing endogenous CXCR7 and AKT expression levels vary among human lung cancer cell lines. ***B*,** H1299-IL24 cells were treated with doxycycline (1 μg/ml). At 24 h and 48 h after treatment, cells were harvested, cell lysates prepared and analyzed for CXCR7 expression by western blotting. Cells that were not treated with doxycycline served as control IL-24 did not reduce CXCR7 expression at 24 h and 48 h when compared to control. Beta actin was used as protein loading control.
(TIF)

S3 Fig Doxycycline alone does not inhibit lung tumor cell migration. The inhibitory activity of doxycycline on migration of naïve H1299 cells was determined by treating the cells with doxycycline (1 μg/ml). Cells that were not treated with doxycycline served as control. No significant inhibitory effect was observed in doxycycline treated H1299 cells when compared to control at all-time points tested.
(TIF)

S4 Fig IL-24 inhibits SDF-1 induced CXCR4 activation. Fluo-4 direct calcium assay showing Ca^2+^ mobilization was inhibited on induction of IL-24 expression in SDF-1 treated H1299-IL24 cells compared to Ca^2+^ mobilization in SDF-1 treated cells that did not express IL-24. IL-24-mediated inhibitory activity however was not statistically significant.
(TIF)

S5 Fig SJA5 shows greater inhibitory effect on CXCR4 expression than AMD3100. Reduction in CXCR4 expression was more pronounced in SJA5 (100 ng/ml) treated cells at all-time points tested compared to CXCR4 expression in AMD3100 treated cells. Additionally, the CXCR4 inhibitory activity exerted by SJA5 appeared to be sustained over time while the inhibitory activity in AMD3100 treatment was gradually lost as evidenced by the increase in CXCR4 expression levels that was approaching the levels observed in untreated control cells. Beta actin was used as protein loading control. Differences in the expression of the proteins was determined by semi-quantitative analysis and represented in graphical format. *P*<0.05 was considered statistically significant.
(TIF)

S6 Fig IL-24 combined with SJA5 exhibited greater inhibitory activity on SDF-1 induced cell migration. Combination of IL-24 and SJA5 resulted in significant suppression of SDF-1 induced cell migration compared to number of cells that migrated in the control group (*P*< 0.05). Additionally, inhibitory activity exerted by IL-24 and SJA5 combination treatment was greater than that observed with other treatment groups. Error bars denote standard deviation.
(TIF)
