# Supplementary figures and images for: IL-24 Inhibits Lung Cancer Cell Migration and Invasion by Disrupting The SDF-1/CXCR4 Signaling Axis
Source: PLoS One. 2015 Mar 16;10(3):e0122439. doi: 10.1371/journal.pone.0122439 (PMC4361489; doi:10.1371/journal.pone.0122439)

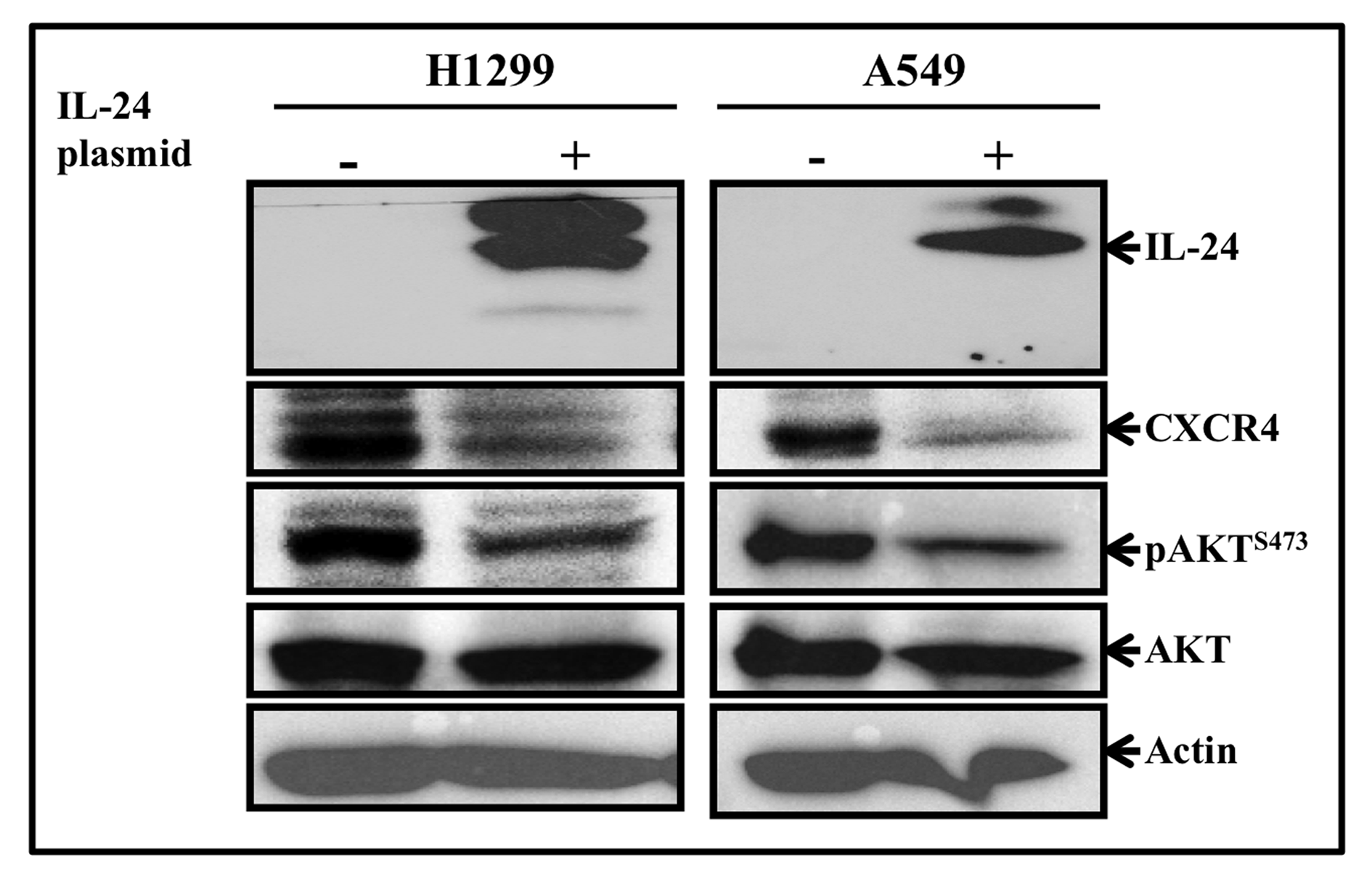

Supplement: S1 Fig — Transient transfection of IL-24 plasmid DNA reduced CXCR4 and pAKTS473 protein expression in both H1299 and A549 cells compared to their respective non-transfected cells. Beta actin was used as protein loading control. (TIF) [file pone.0122439.s001.tif]

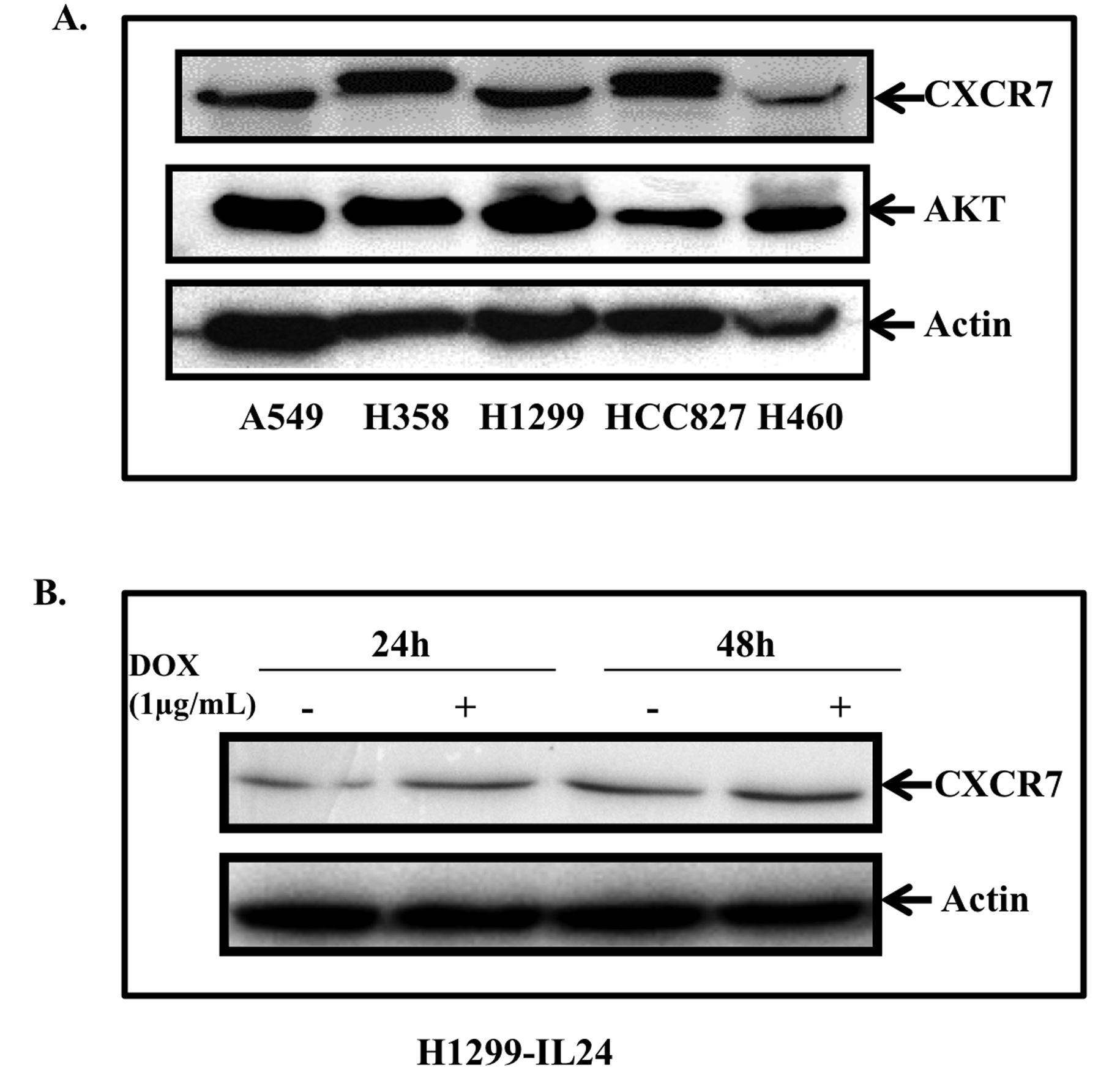

Supplement: S2 Fig — A, Western blotting showing endogenous CXCR7 and AKT expression levels vary among human lung cancer cell lines. B, H1299-IL24 cells were treated with doxycycline (1 μg/ml). At 24 h and 48 h after treatment, cells were harvested, cell lysates prepared and analyzed for CXCR7 expression by western blotting. Cells that were not treated with doxycycline served as control IL-24 did not reduce CXCR7 expression at 24 h and 48 h when compared to control. Beta actin was used as protein loading control. (TIF) [file pone.0122439.s002.tif]

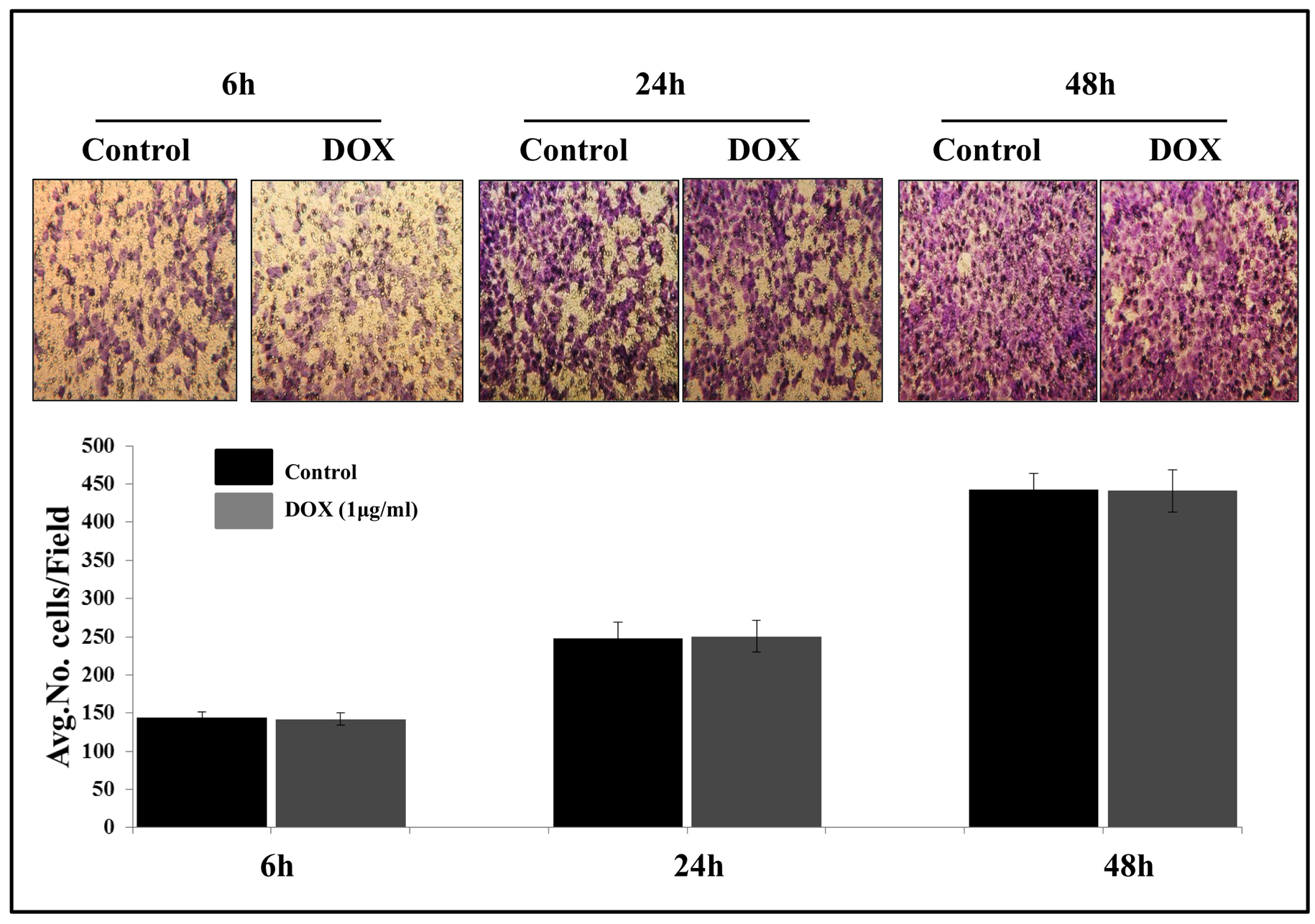

Supplement: S3 Fig — The inhibitory activity of doxycycline on migration of naïve H1299 cells was determined by treating the cells with doxycycline (1 μg/ml). Cells that were not treated with doxycycline served as control. No significant inhibitory effect was observed in doxycycline treated H1299 cells when compared to control at all-time points tested. (TIF) [file pone.0122439.s003.tif]

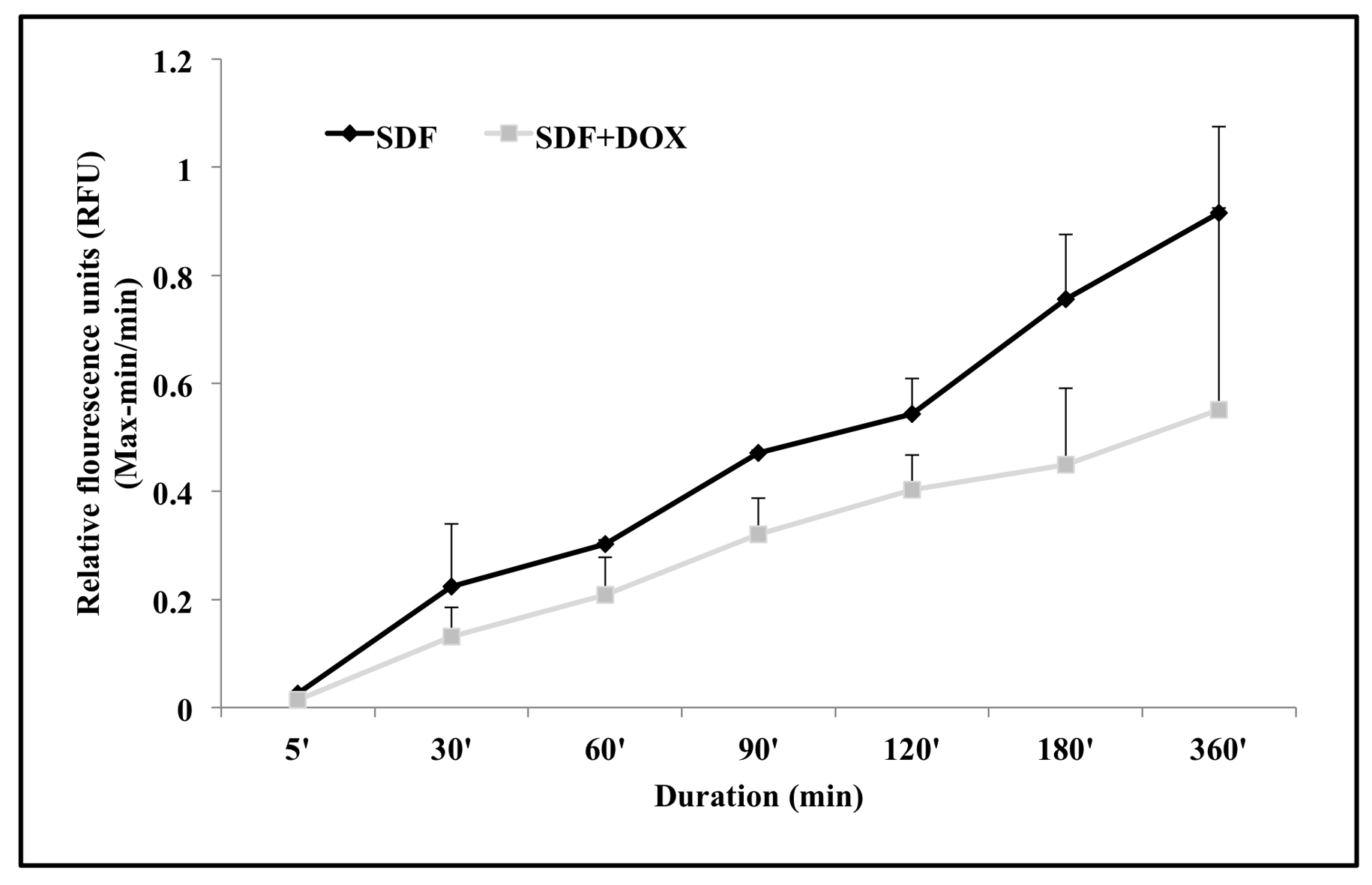

Supplement: S4 Fig — Fluo-4 direct calcium assay showing Ca2+ mobilization was inhibited on induction of IL-24 expression in SDF-1 treated H1299-IL24 cells compared to Ca2+ mobilization in SDF-1 treated cells that did not express IL-24. IL-24-mediated inhibitory activity however was not statistically significant. (TIF) [file pone.0122439.s004.tif]

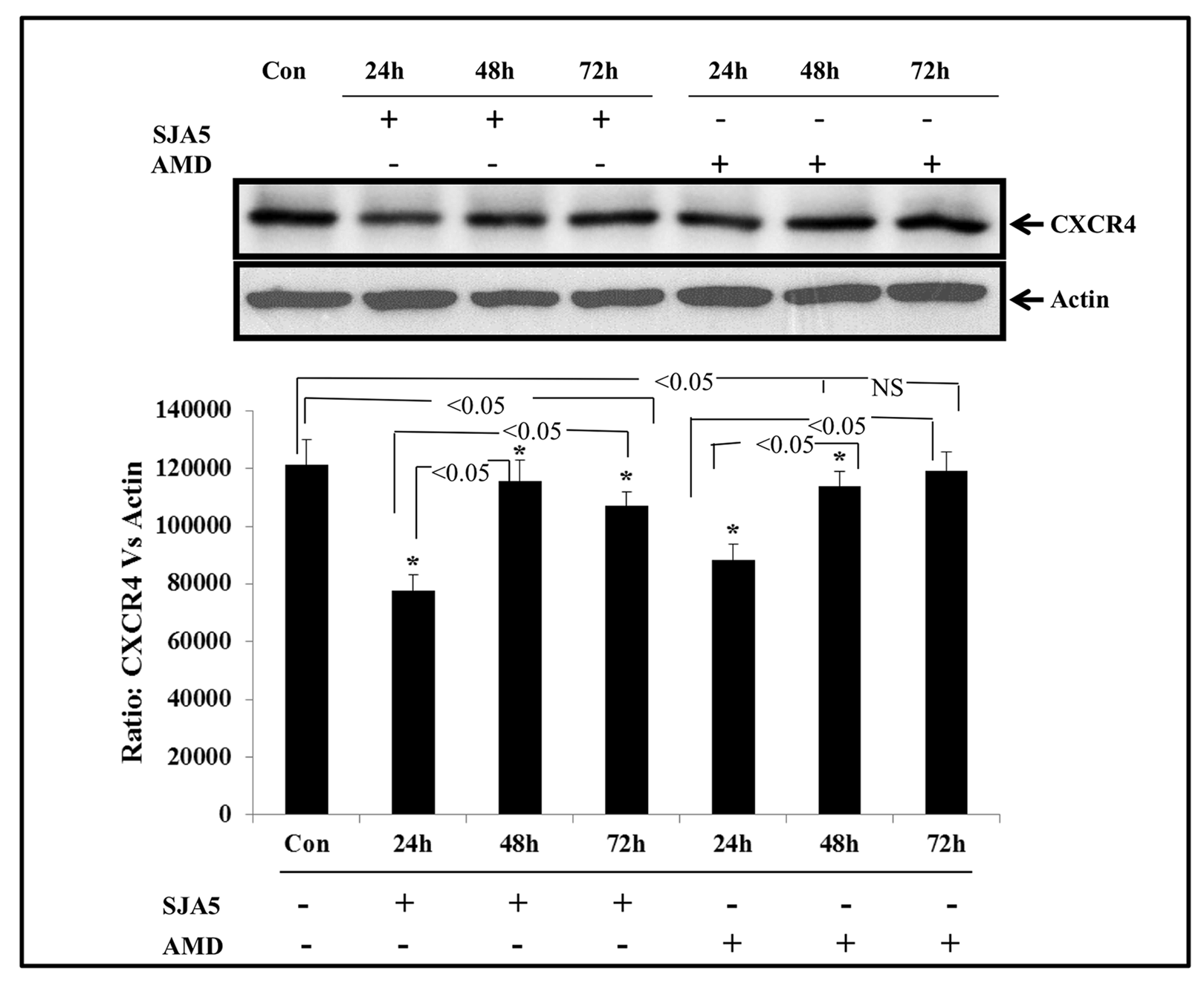

Supplement: S5 Fig — Reduction in CXCR4 expression was more pronounced in SJA5 (100 ng/ml) treated cells at all-time points tested compared to CXCR4 expression in AMD3100 treated cells. Additionally, the CXCR4 inhibitory activity exerted by SJA5 appeared to be sustained over time while the inhibitory activity in AMD3100 treatment was gradually lost as evidenced by the increase in CXCR4 expression levels that was approaching the levels observed in untreated control cells. Beta actin was used as protein loading control. Differences in the expression of the proteins was determined by semi-quantitative analysis and represented in graphical format. P<0.05 was considered statistically significant. (TIF) [file pone.0122439.s005.tif]

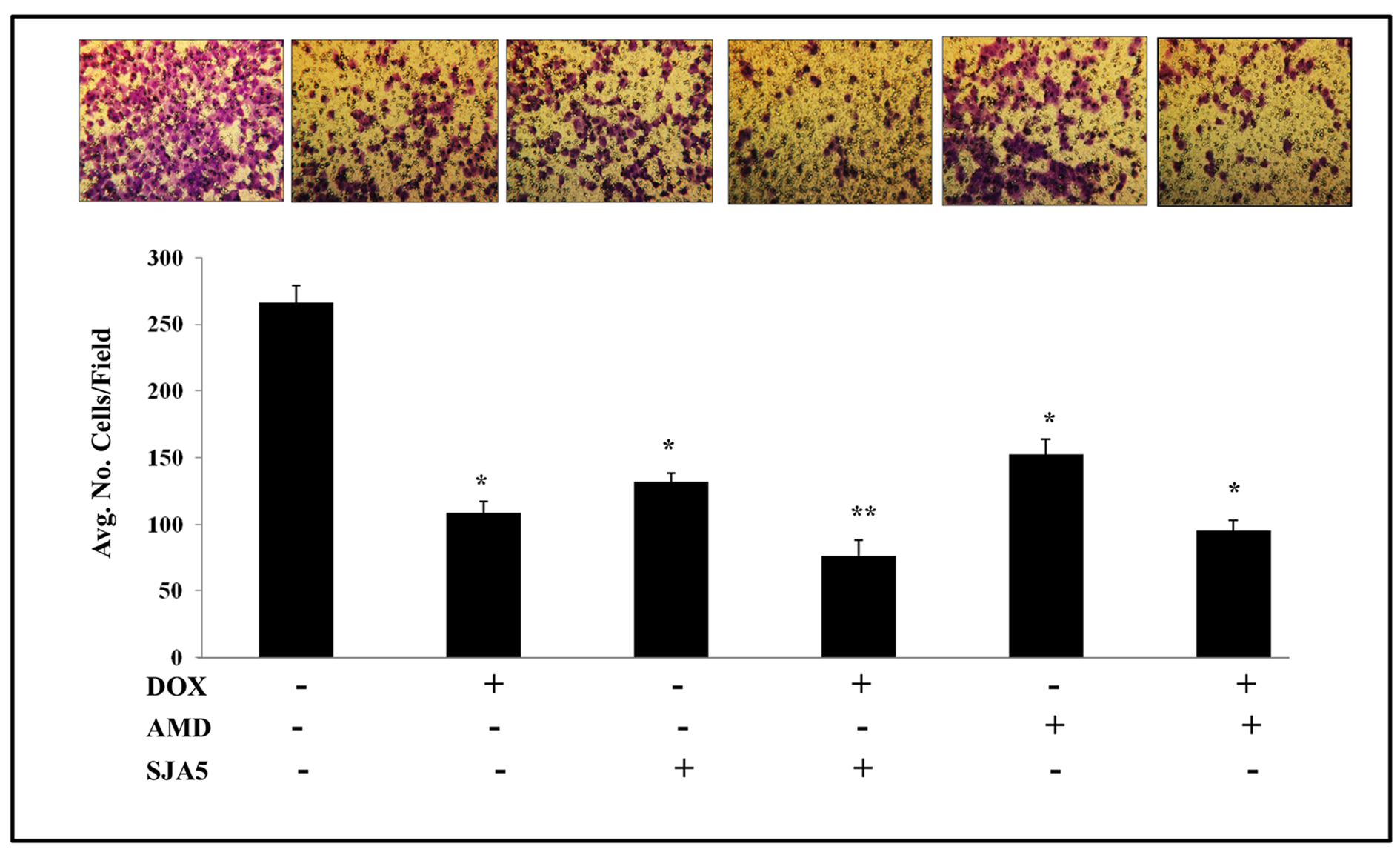

Supplement: S6 Fig — Combination of IL-24 and SJA5 resulted in significant suppression of SDF-1 induced cell migration compared to number of cells that migrated in the control group (P< 0.05). Additionally, inhibitory activity exerted by IL-24 and SJA5 combination treatment was greater than that observed with other treatment groups. Error bars denote standard deviation. (TIF) [file pone.0122439.s006.tif]
